# Proposal and Assessment of a De-Identification Strategy to Enhance Anonymity of the Observational Medical Outcomes Partnership Common Data Model (OMOP-CDM) in a Public Cloud-Computing Environment: Anonymization of Medical Data Using Privacy Models

**DOI:** 10.2196/19597

**Published:** 2020-11-26

**Authors:** Seungho Jeon, Jeongeun Seo, Sukyoung Kim, Jeongmoon Lee, Jong-Ho Kim, Jang Wook Sohn, Jongsub Moon, Hyung Joon Joo

**Affiliations:** 1 Division of Information Security Graduate School of Information Security Korea University Seoul Republic of Korea; 2 Korea University Research Institute for Medical Bigdata Science Korea University Seoul Republic of Korea; 3 Department of Cardiology, Cardiovascular Center Korea University Seoul Republic of Korea; 4 Division of Infectious Diseases Department of Internal Medicine, College of Medicine Korea University Seoul Republic of Korea; 5 Department of Internal Medicine Korea University College of Medicine Korea University Seoul Republic of Korea

**Keywords:** de-identification, privacy, anonymization, common data model, Observational Health Data Sciences and Informatics

## Abstract

**Background:**

De-identifying personal information is critical when using personal health data for secondary research. The Observational Medical Outcomes Partnership Common Data Model (CDM), defined by the nonprofit organization Observational Health Data Sciences and Informatics, has been gaining attention for its use in the analysis of patient-level clinical data obtained from various medical institutions. When analyzing such data in a public environment such as a cloud-computing system, an appropriate de-identification strategy is required to protect patient privacy.

**Objective:**

This study proposes and evaluates a de-identification strategy that is comprised of several rules along with privacy models such as k-anonymity, l-diversity, and t-closeness. The proposed strategy was evaluated using the actual CDM database.

**Methods:**

The CDM database used in this study was constructed by the Anam Hospital of Korea University. Analysis and evaluation were performed using the ARX anonymizing framework in combination with the k-anonymity, l-diversity, and t-closeness privacy models.

**Results:**

The CDM database, which was constructed according to the rules established by Observational Health Data Sciences and Informatics, exhibited a low risk of re-identification: The highest re-identifiable record rate (11.3%) in the dataset was exhibited by the DRUG_EXPOSURE table, with a re-identification success rate of 0.03%. However, because all tables include at least one “highest risk” value of 100%, suitable anonymizing techniques are required; moreover, the CDM database preserves the “source values” (raw data), a combination of which could increase the risk of re-identification. Therefore, this study proposes an enhanced strategy to de-identify the source values to significantly reduce not only the highest risk in the k-anonymity, l-diversity, and t-closeness privacy models but also the overall possibility of re-identification.

**Conclusions:**

Our proposed de-identification strategy effectively enhanced the privacy of the CDM database, thereby encouraging clinical research involving multiple centers.

## Introduction

The Observational Medical Outcomes Partnership Common Data Model (OMOP-CDM), defined by the nonprofit organization Observational Health Data Sciences and Informatics (OHDSI) [1], is a standard data schema [2,3] that uses standardized terms [4]. An established CDM database can be used by multiple institutions to conduct quick analyses under the same conditions using the analysis tools provided by OHDSI, such as Atlas [5] and Achilles [6]. Furthermore, CDM can be used not only for research in combination with a distributed research network for collecting results analyzed by an individual institution but also in clinical decision support systems for patient-specific medical treatment by combining advanced analysis and prediction techniques such as artificial intelligence. Consequently, many medical institutions have recently attempted establishing CDM databases.

However, owing to security concerns, existing CDM databases remain inaccessible from outside the network of their institutions; therefore, only authorized researchers are allowed to analyze their clinical data. Moreover, institutions operate the CDM databases only in on-premise environments for security concerns regarding sensitive information. Nevertheless, owing to increasing system complexities and service availability, operators generally prefer to run the CDM database in a cloud-computing environment. This recent trend has led to recent regulatory and legal considerations regarding network accessibility [7].

Meanwhile, while the demand for research using medical data that has been accumulated over the past decades has increased, interinstitutional research specifically requires appropriate de-identification of medical data because of privacy concerns. Hence, to address these concerns, anonymization studies and frameworks are currently being proposed for various datasets [8-10].

As CDM database research does not extract nor analyze the institutional raw data, it involves a low risk of personal information disclosure. However, as various clinical databases are accessed and analyzed in a public environment, the construction of a CDM database requires not only an access control policy but also highly tailored protection mechanisms in addition to evaluation of the adequacy of the anonymization [11]. Therefore, in this study, we proposed and evaluated a de-identification strategy for the OMOP-CDM database using privacy models such as k-anonymity.

## Methods

### OMOP-CDM Database

The OMOP-CDM database is one of the significant core projects managed by the OHDSI, which uses common representation for the clinical data of various projects to support medical research. For instance, ATHENA is a standardized vocabulary [4], Atlas is a unified web-based interface to analyze CDM data [5], and Achilles is used for data characterization [6]; in addition to these tools, several useful applications have also been provided [12-14].

The specifications of the OMOP-CDM [15] are actively being amended according to the needs of researchers. At the time of writing this article, version 6.0.0 of the CDM had been published. However, this research uses the CDM database constructed by the Korea University Anam Hospital on March 23, 2020, based on version 5.3.1 of the OMOP-CDM schema. The tables comprising the database include the standardized vocabulary, metadata, clinical data tables, health system data tables, health economics, and derived elements, in addition to the results schema.

The standardized vocabulary contains 10 tables with detailed information on the concepts used in the OMOP-CDM, while the standardized metadata preserves the entire metadata information derived from data that have been transformed into the OMOP-CDM database in 2 tables. Similarly, the 16 standardized clinical tables contain patient clinical data; the tables that store the clinical data have a relation with the PERSON table, which stores the patient’s personal information; the 4 standardized health system data tables contain information on the agency providing the treatment; the 2 standardized health economics data tables contain payment information for the medical services; the standardized derived elements store information such as the dosing period in 3 tables; and the results schema stores information such as the definition of each cohort in 2 tables. In the OMOP-CDM, some raw source data collected by institutions remain in fields named “source_value,” which are used for flexibility and research convenience and are generally not shared outside the source institution. However, because of the high accessibility of the cloud-computing environment, these should be protected using proper security measures.

### ARX Anonymization Framework

This study uses the ARX anonymization framework [16] as the evaluation tool for de-identification. ARX is awidely used open-source software for anonymizing data that include sensitive information; it supports a variety of privacy models, risk analysis models, data transformation methods, and methods to analyze the anonymized data.

The anonymizing process using ARX can be divided into 4 steps: (1) configuration, (2) exploration, (3) utility analysis, and (4) risk analysis. In the configuration step, ARX imports the data and then assigns each field to one of the following elements: identifier, quasi-identifier, sensitive data, or insensitive data. The information in the fields that are set as identifiers is removed in the anonymizing process. Transformation rules such as generalization or aggregation are applied to the data in the fields that are set as quasi-identifiers: The fields marked as sensitive or insensitive data do not undergo transformation, and the sensitive fields are protected by a privacy model such as k-anonymity. Once the field attribution is complete, ARX creates the privacy model and sets the model parameters before finally performing the data anonymization. In addition, ARX represents possible transformations as a lattice and can produce various levels of data anonymization depending on the transformation rules and the selected privacy model. In the exploration step, the user explores the results and selects the transformation for the analysis. In the utility analysis step, the user evaluates the transformation selected in the previous step by various statistical analysis methods such as logistic regression and chooses a suitable transformation for the de-identification scenario expected by the user. Finally, in the risk analysis step, ARX analyzes the risk of re-identification under the chosen transformation using 3 re-identification attacker models: prosecutor, journalist, and marketer [17]. In the prosecutor attacker model, the attacker is assumed to know the targeted individual in the database. In the journalist attacker model, an attacker does not know whether the targeted person is listed in the database. In the marketer attacker model, an attacker aims at identifying a large number of records from a database, rather than any particular individual. This study excluded the CDM tables that did not undergo the extract-transform-load (ETL) procedure from the evaluation process and randomly sampled the data from the database for tables exceeding the maximum analytical data size of ARX (*rows* x *cols* =2^32^ – 1).

### Methods for Anonymizing Personally Identifiable Information

Anonymization techniques for personally identifiable information include pseudonymization, aggregation, data reduction, categorization, and masking. Pseudonymization is the generic term for replacing personally identifiable information with other values; it includes heuristic pseudonymization, encryption, and substitution. In aggregation, an individual value is replaced with a statistical value such as the mean or median of the identifiers or quasi-identifiers of the group to which the individual belongs. Data reduction is the simple removal of elements that make an individual identifiable: Although this is the strongest anonymizing method, it causes loss of some important data and degrades the value of the entire dataset. Categorization is the most common anonymization method, wherein personal information is replaced with a representative group value. Finally, masking converts a portion of the data to either a blank or noise, such as “*”.

## Results

### Proposal for De-Identification of Personal Information in CDM

Owing to the importance of de-identifying personal information while using personal health data for secondary research, the OMOP-CDM already implements a certain level of de-identification during the construction of its database: The reference architecture provided by OHDSI, namely the OHDSIonAWS [18], uses several anonymization methods to comply with the Health Information Portability and Accountability Act (HIPAA) [7]. However, although the OMOP-CDM adopts standardized terminology, it is designed to maintain the original source information in the field of “_SOURCE_VALUE.” Therefore, it is necessary to consider the appropriateness of the de-identification level for this database. In this study, we propose an enhanced de-identification strategy that is comprised of a set of rules for privacy models such as k-anonymity, l-diversity, and t-closeness for the OMOP-CDM from the perspective of reconnection with other information and privacy models.

The de-identification strategy for the CDM presented in this research is as follows: First, according to the Safe Harbor method in the HIPAA privacy rule [19], the data included in the CDM are classified as identifiers, quasi-identifiers, raw data, sensitive data, or insensitive data. “Raw data” refers to fields including the keyword “source_value” in the OMOP-CDM convention: A raw data field preserves data from the institution’s database before it is converted to the CDM database through ETL. Second, the identifiers are deleted, and the raw data are protected by anonymization methods; specifically, the foreign key that can be linked with the institutional database is deleted. Third, fields containing intentionally stored data such as those in the SURVEY_CONDUCT table are maintained. Fourth, if a field includes identifiers or quasi-identifiers, this field or table must not be used, although it is intentionally stored.

Table 1 shows the transformations for the fields named “source_value.” These rules collaborate with the privacy models to achieve a high de-identification level. The table lists only the fields named by the “source_value” or those possibly containing (quasi-)identifiers.

Identifiers such as the name and social security number are removed during the ETL. We designated the fields in Table 1 as quasi-identifiers because they contain raw data. However, the fields containing clinical data such as “quantity” in DRUG_EXPOSURE are considered as sensitive information and are therefore protected by anonymizing the quasi-identifiers using either the l-diversity or t-closeness model.

In Table 1, most tables such as PERSON and OBSERVATION clearly contain sensitive information that may identify a specific individual. However, the data stored in the LOCATION, CARE_SITE, PROVIDER, PAYER_PLAN, and COST fields do not directly identify the individual: For instance, the CARE_SITE table contains information regarding the institution at which health care has been delivered. Despite its property, this information may be sensitive because a combination of these data could implicitly specify the individual. However, following the proposed strategy could reduce the number of data records. For instance, even after de-identification is implemented, if the combination of the identifiers and quasi-identifiers is unique or does not satisfy the pre-defined criteria of the privacy models (ie, “k,” “l,” or “t”), this data could identify the individual in the database. Although there seems to be a loss of important data, this strategy actually minimizes the impact on the data analyses from de-identification because the “source_value” fields can be replaced by the “source_concept_id” fields. For instance, the “gender_source_value” of the PERSON table stores the gender of the individuals as recorded in the institution: For example, this value could be “Male/Female” or “0/1”; the standard concept for gender in OMOP-CDM is “M/F.”

**Table 1:** Proposal for enhanced de-identification for the Observational Medical Outcomes Partnership Common Data Model (OMOP-CDM) specification. Information regarding the CDM table, its fields, and its descriptions have been cited from [15].

Tables and fields			Description		De-identification method
**PERSON**					
	Person_source_value	A key derived from a personal identifier in the source data		Remove^a^	
	Gender_source_value, Race_source_value, Ethnicity_source_value	The codes for the gender, race, and ethnicity of an individual as they appear in the source data		Masking^b^	
**VISIT_OCCURRENCE**					
	Visit_source_value, Admitting_from_source_value, Discharge_to_source_value	The codes for the visit, where the patient was admitted from, and the discharge disposition as they appear in the source data		Masking	
**VISIT_DETAIL**					
	Visit_detail_source_value, Admitted_from_source_value, Discharge_to_source_value	The codes for the visit, admitting source, discharge disposition, and optional details information as they appear in the source data		Masking	
**CONDITION_OCCURRENCE**					
	Condition_source_value, Condition_status_source_value	The codes for the condition and its status as they appear in the source data		Masking	
**DRUG_EXPOSURE**					
	Sig	The directions on the drug prescription as recorded in the original prescription		NLP^c^	
	Drug_source_value, Route_source_value, Dose_unit_source_value	The codes for the drug, administration route, and dose unit as they appear in the source data		Masking	
**PROCEDURE_OCCURRENCE**					
	Procedure_source_value, Modifier_source_value	The codes for the procedure and qualifier as they appear in the source data		Masking	
**DEVICE_EXPOSURE**					
	Device_source_value	The code for the device as it appears in the source data		Masking	
**MEASUREMENT**					
	Measurement_source_value, Unit_source_value, Value_source_value	The measurement name, unit (code), and value as a number as they appear in the source data		Masking	
**NOTE**					
	Note_text	The content of the note		NLP	
**SURVEY_CONDUCT**					
	Validated_survey_source_value, Survey_source_identifier	Source value representing the validation status of the survey and a unique identifier for each completed survey in the source system		Masking	
**OBSERVATION**					
	Value_as_string	The observation result stored as a string		NLP	
**SPECIMEN**					
	Specimen_source_value, Unit_source_value, Anatomic_site_source_value, Disease_status_source_value	The specimen value, unit information, anatomic site, and disease status information as they appear in the source data		Masking	
**LOCATION**					
	Address_1, address_2	The address field (street address, building, suite, floor) and zip or postal code as they appear in the source data		Partial remove^d^	
	Latitude, longitude	The geocoded latitude and longitude		Remove	
	Location_source_value	The information that is used to uniquely identify the location as it appears in the source data		Masking	
**CARE_SITE**					
	Care_site_source_value	The identifier for the care site and the source code for the Place of Service in the source data		Masking	
**PROVIDER**					
	Provider_name, npi, dea, Provider_source_value, Specialty_source_value, Gender_source_value	The provider name, National Provider Identifier (NPI), Drug Enforcement Administration (DEA) number of the provider, provider identifier, source code for the provider specialty, and gender information in the source data		Masking	
**PAYER_PLAN_PERIOD**					
	Payer_source_value, Plan_source_value, Contract_source_value, Sponsor_source_value, Family_source_value	The source codes for the payer, health benefit plan, reason justifying the contract, sponsor of the health plan, family, and reason for stopping coverage as they appear in the source data		Masking	
**COST**					
	Cost_source_value, Revenue_code_source_value, Drg_source_value	The source values for the cost, revenue code, and three-digit drug source code as they appear in the source data		Masking	

^a^Refers to the deletion of the entire value stored in the field.

^b^Refers to the replacement of a part of the value with another character such as “*”.

^c^NLP: natural language processing.

^d^Refers to deletion of only a part of the value.

### Evaluation and Validation of De-Identification of Personal Information in CDM

The aforementioned principles apply to identifiers, quasi-identifiers, and raw data. However, personal information that is often disclosed as sensitive information must also be protected via an appropriate de-identification policy according to the laws in force like HIPAA as well as the adopted privacy models. As already mentioned, the commonly used privacy models include k-anonymity [20], l-diversity [21], and t-closeness [22], among which k-anonymity is most widely used. In this model, anonymization methods are first applied to the identifiers and quasi-identifiers. Then, all records are divided into groups such that each group includes all records with identical anonymized identifiers and quasi-identifiers; a group containing fewer than *k* records is discarded. Therefore, the probability of identifying an individual is *1/k*. However, if the anonymization is applied only to the identifiers and quasi-identifiers and the sensitive information is not hidden or anonymized, then an individual can be identified using the personal information that is already known — this is called a homogeneity attack. To reduce the risk of such attacks, the l-diversity model is used, which further divides the groups in the k-anonymity model such that the elements in each group have at least *l* different values for the sensitive data; groups with fewer than *l* elements are deleted. Although the application of this model can improve the de-identification level, some individual information exposure is still possible: For instance, if the distribution of sensitive data in each group is significantly skewed (ie, the sensitive data hold biased information in a particular space [or domain]), it is possible to deduce that an individual is associated with that domain — this is called a skewness attack. Therefore, to protect against such attacks, the t-closeness model is introduced, which forces domain distribution between groups of less than or equal to a pre-defined *t*. In this model, the distribution of the sensitive data in each group occurs between similar areas. The choice of the appropriate privacy model can be made by considering the value of the dataset and impact in the event of data disclosure.

As all 3 privacy models essentially achieve their security goals by anonymizing the identifiers and quasi-identifiers, the personal information administrator should appropriately designate identifiers or quasi-identifiers in a dataset following the institutional security policy.

In this research, we set the identifiers and quasi-identifiers to establish the transformation rules according to the proposed strategy to improve the de-identification level of the OMOP-CDM. The following is a detailed explanation: The proposed de-identification strategy can be achieved by using one of the privacy models combined with the rules described in Table 1. If the administrator chooses the k-anonymity privacy model, the fields in Table 1 are considered as quasi-identifiers and anonymized to stand for a pre-defined group of size k. However, if the l-diversity or t-closeness model is chosen, the fields in Table 1 are regarded as quasi-identifiers like in the former case, while the other fields containing clinical data, such as “quantity” in DRUG_EXPOSURE, are designated as sensitive data. Then, the anonymizing process proceeds for quasi-identifiers while complying with pre-defined “l” or “t.”

Table 2 presents the number of data values for each table included in the CDM database established by the Korea University Anam Hospital that was used for the evaluation, wherein we analyzed the re-identification risk for the individuals in the PERSON table as well as those joined to the PERSON table through the other tables including the patient-level clinical data. While anonymizing personal information using ARX, each field in the table should be designated as the identifier, quasi-identifier, sensitive data, or insensitive data. As the identifier has already been removed from the OMOP-CDM schema during the ETL process, each field in the table is classified as 1 of the 3 remaining types. In our proposed strategy, the raw data are considered as quasi-identifiers and are therefore anonymized by the transformation rules when privacy models, such as k-anonymity, l-diversity, and t-closeness, are applied. In this evaluation, the fields listed in Table 1 were designated as quasi-identifiers, while the remaining fields, including patient-level clinical data other than the PERSON table, were designated as sensitive data.

**Table 2:** Number of data values per table in the Korea University Anam Hospital Common Data Model (CDM) database.

Tables in the database	Number of values
PERSON	1,891,755
CONDITION_OCCURRENCE	28,704,247
CONDITION_ERA	14,972,790
DEVICE_EXPOSURE	33,617,896
DOSE_ERA	64,047,133
DRUG_ERA	29,274,258
DRUG_EXPOSURE	77,919,053
MEASUREMENT	196,567,735
OBSERVATION	1,744,021
OBSERVATION_PERIOD	1,629,356
PROCEDURE_OCCURRENCE	21,200,346

Tables 3, 4, and 5 present the results of the re-identification risk analysis when the k-anonymity, l-diversity, and t-closeness privacy models, respectively, are applied to the CDM database. This means that the presented de-identification strategy is applied for the fields containing the source data in each table after ETL. In these tables, the re-identification risk indicates that some combinations of source data uniquely appear in the CDM database; this further implies re-identification for the individual. The ARX supports 3 re-identification attacker models for the re-identification risk analysis, namely the prosecutor, journalist, and marketer models. Herein, we used the prosecutor model, which analyzes the possibility of an attacker identifying a person under the assumption that they are already aware that the person is included in the dataset. Some fields requiring natural language processing (NLP) have been removed because ARX does not support the anonymizing techniques for free text. Furthermore, even if free text is appropriately anonymized, it is likely to have unique values, which make it difficult to meet the criteria of the privacy models used in this study. The tables present the results of applying the optimal transformation suggested by ARX. In accordance with the applicable national guidelines for de-identification of personal information [23], k-anonymity was applied with the minimum criterion, *k* = 3, while the remaining models used the most conservative conditions, guaranteeing stronger anonymity than the k-anonymity model (ie, *l* = 5, *t*= 0.1). For instance, if *l* is <5 in the l-diversity model applied to this dataset, ARX suggests a level of transformation equal to that of k-anonymity for most of the tables.

**Table 3:** Re-identification risks before and after source_value anonymization (k-anonymity, k = 3).

Tables		Records at risk^a^ (%)	Highest risk^b^ (%)	Success rate^c^ (%)
**PERSON**				
	Before^d^	0.1	100	0.11
	After^e^	0	1.27	0.01
**CONDITION_OCCURRENCE**				
	Before	7.58	100	6.39
	After	<0.01	25	<0.01
**CONDITION_ERA**				
	Before	0.02	100	0.03
	After	0	6.25	<0.01
**DEVICE_EXPOSURE**				
	Before	6.98	100	5.73
	After	0	0.3	<0.01
**DOSE_ERA**				
	Before	0.02	100	0.03
	After	<0.01	25	<0.01
**DRUG_ERA**				
	Before	0.02	100	0.03
	After	0	16.67	<0.01
**DRUG_EXPOSURE**				
	Before	11.31	100	8.86
	After	<0.01	33.33	<0.01
**MEASUREMENT**				
	Before	1.53	100	1.56
	After	0	6.25	<0.01
**OBSERVATION**				
	Before	1.63	100	1.55
	After	0	0.98	<0.01
**OBSERVATION_PERIOD**				
	Before	0.11	100	0.12
	After	0	6.25	<0.01
**PROCEDURE_OCCURRENCE**				
	Before	4.25	100	3.7
	After	0	0.37	<0.01

^a^Represents the percentage of data in the dataset that exceeds the risk threshold.

^b^Represents the highest risk for an individual data value.

^c^Percentage of data that can be re-identified in the dataset on average.

^d^Refers to before applying the anonymizing methods to the data.

^e^Refers to after applying the anonymizing methods to the data.

**Table 4:** Re-identification risks before and after source_value anonymization (l-diversity, l = 5).

Tables		Records at risk^a^ (%)	Highest risk^b^ (%)	Success rate^c^ (%)
**CONDITION_OCCURRENCE**				
	Before^d^	7.58	100	6.39
	After^e^	0	0.36	<0.01
**CONDITION_ERA**				
	Before	0.02	100	0.03
	After	0	0.38	<0.01
**DEVICE_EXPOSURE**				
	Before	6.98	100	5.73
	After	0	0.3	<0.01
**DOSE_ERA**				
	Before	0.02	100	0.03
	After	0	0.2	<0.01
**DRUG_ERA**				
	Before	0.02	100	0.03
	After	0	0.2	<0.01
**DRUG_EXPOSURE**				
	Before	11.31	100	<8.86
	After	0	0.19	<0.01
**MEASUREMENT**				
	Before	1.53	100	1.56
	After	0	<0.01	<0.01
**OBSERVATION**				
	Before	1.63	100	1.55
	After	0	0.08	0.01
**OBSERVATION_PERIOD**				
	Before	0.11	100	0.12
	After	0	0.58	<0.01
**PROCEDURE_OCCURRENCE**				
	Before	4.25	100	3.7
	After	0	0.37	<0.01

^a^Represents the percentage of data in the dataset that exceeds the risk threshold.

^b^Represents the highest risk for an individual data value.

^c^Percentage of data that can be re-identified in the dataset on average.

^d^Refers to before applying the anonymizing methods to the data.

^e^Refers to after applying the anonymizing methods to the data.

**Table 5:** Re-identification risks before and after source_value anonymization (t-closeness, *t*= 0.1).

Tables		Records at risk^a^ (%)	Highest risk^b^ (%)		Success rate^c^ (%)	
**CONDITION_OCCURRENCE**						
	Before^d^	7.58		100		6.39
	After^e^	0		<0.01		<0.01
**CONDITION_ERA**						
	Before	0.02		100		0.03
	After	0		<0.01		<0.01
**DEVICE_EXPOSURE**						
	Before	6.98		100		5.73
	After	0		<0.01		<0.01
**DOSE_ERA**						
	Before	0.02		100		0.03
	After	0		<0.01		<0.01
**DRUG_ERA**						
	Before	0.02		100		0.03
	After	0		<0.01		<0.01
**DRUG_EXPOSURE**						
	Before	11.31		100		8.86
	After	0		<0.01		<0.01
**MEASUREMENT**						
	Before	1.53		100		1.56
	After	0		<0.01		<0.01
**OBSERVATION**						
	Before	1.63		100		1.55
	After	0		<0.01		<0.01
**OBSERVATION_PERIOD**						
	Before	0.11		100		0.12
	After	0		<0.01		<0.01
**PROCEDURE_OCCURRENCE**						
	Before	4.25		100		3.7
	After	0		<0.01		<0.01

^a^Represents the percentage of data in the dataset that exceeds the risk threshold.

^b^Represents the highest risk for an individual data value.

^c^Percentage of data that can be re-identified in the dataset on average.

^d^Refers to before applying the anonymizing methods to the data.

^e^Refers to after applying the anonymizing methods to the data.

As presented in the analysis results, before applying the de-identification strategy (ie, immediately after the ETL), the CDM database exhibits a significantly low possibility of re-identification. The DRUG_EXPOSURE table presented the highest percentage of records at risk, at 11.31% (8,812,644 records), and an average re-identification attack success rate of 8.86% (6,903,628 records) through a combination of the source values. The DOSE_ERA table yielded the lowest percentage of records at risk, at 0.02% (12,809 records), and an average re-identification attack success rate of 0.03% (19,214 records). Although the OMOP-CDM already had a high level of anonymity, it exhibited the highest risk (100%) for re-identifiable data in all tables. In other words, there is a risk of an individual being identified solely by a combination of the “source values.” Therefore, a de-identification strategy for the “source value” fields is required for the safe utilization of the CDM data in a public environment such as a cloud-computing system. In fact, in every case where the privacy model was applied, the “highest risks” were substantially reduced, and the overall re-identification possibility was also reduced. Among the privacy models, the k-anonymity model demonstrated the weakest de-identification level, while the t-closeness model yielded the strongest de-identification results. These results are expected because l-diversity and t-closeness models are stronger and complement the k-anonymity model, as we had set conservatively stronger configurations for these 2 models.

### Impact of De-Identification on Data Analysis

We conducted several experiments to empirically observe the impact of the proposed de-identification strategy on the analysis: The analysis codes generated by Atlas were used in these experiments. We arbitrarily selected 2 CDM-based studies published on the internet [24,25] to evaluate the proposed de-identification strategy. All parameters of the 2 OMOP-CDM databases used in the experiments were set to the same values, except for the data to be de-identified.

**Figure 1:**
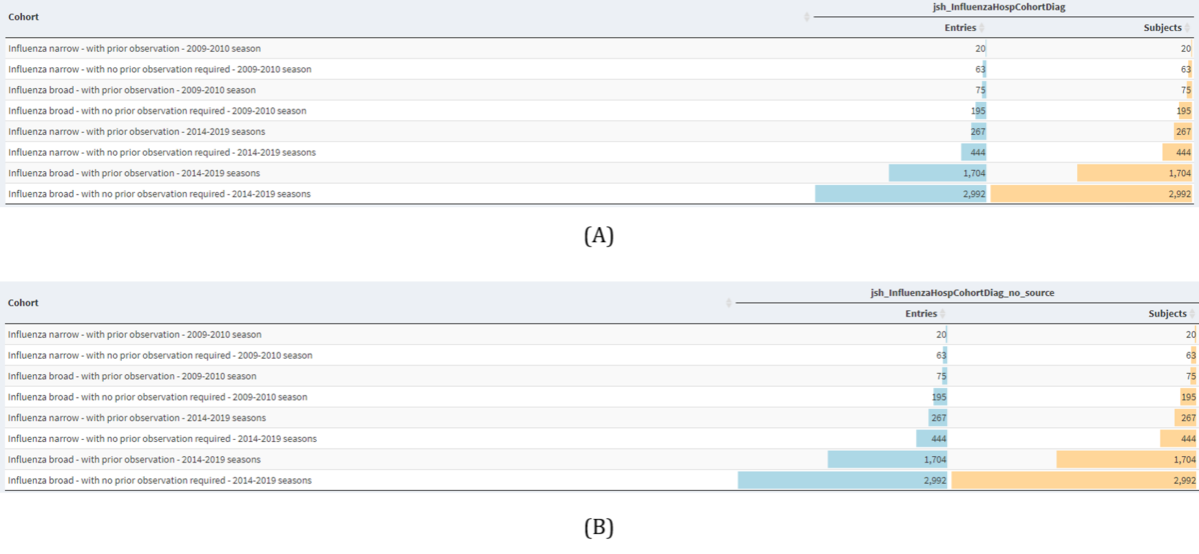
Generated cohorts for “Influenza Cohort Diagnostics” study (A) without de-identification and (B) with the proposed de-identification strategy.

Figure 1 shows the cohorts defined in the “Influenza Cohort Diagnostics” [24] in the “Covid19Hospitalization Characterization” study [26] before and after the proposed de-identification. This study describes the baseline characteristics of influenza (H1N1) patients between 2009-2010 and 2014-2019, builds several cohorts, and systematically presents the characteristics of patients with influenza according to age or gender. Obviously, the same cohorts were generated according to the same definitions before and after the de-identification. This study presents the results of the time distribution as well as the cohort characteristics and their comparison. Furthermore, for convenience, not all analysis results have been presented; however, all metrics showed the exact same values, suggesting that the de-identification of the “source_value” fields does not affect the analysis.

**Figure 2:**
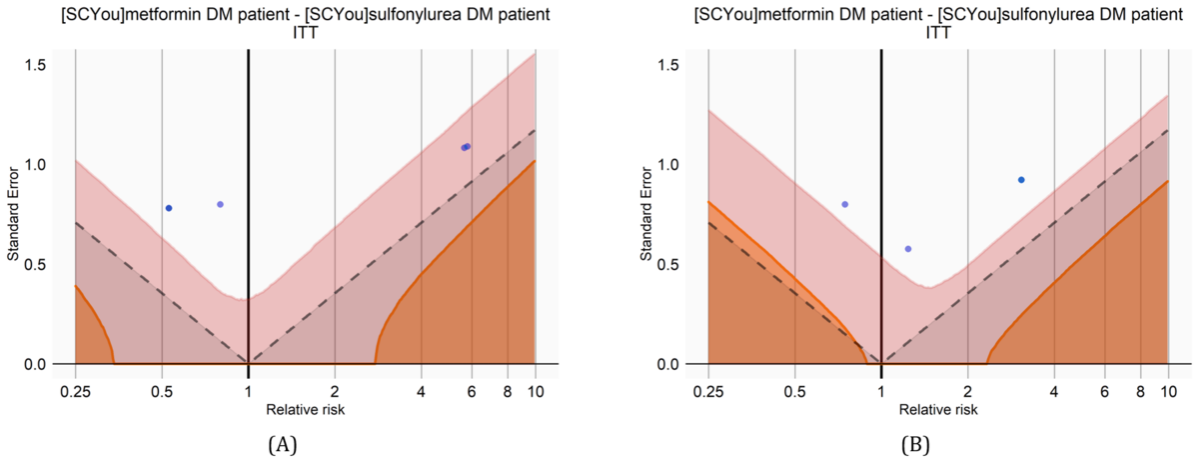
Null distributions for the “MetforminVsSulfonylurea” study (A) without de-identification and (B) with the proposed de-identification strategy. ITT: intention-to-treat.

We also reproduced the “MetforminVsSulfonylurea” study [25], which compares the risk of hypoglycemia among the users of metformin and sulfonylurea. Figure 2 shows the null distributions created in this study: The blue points in the figure represent the estimates of the negative control group on the log scale. Any estimates below the gray dashed lines (gray area) have a conventional *P* value <.05; the shaded orange area estimates have a calibrated *P* value <.05; and the pink area represents the 95% CI. In this case, every estimate from negative controls (dots) has a *P* value >.05 for both conventional and calibrated methods. In this analysis, although slightly different null distributions were generated for every iteration, this study commonly exhibited an area under the curve value of 1 with or without de-identification. Furthermore, we observed that the “source_concept_id” fields were used instead of the “source_value” fields in the SQL queries of this analysis.

Thus, these 2 experiments show that anonymization of the “source_value” fields can not only de-identify individuals in the CDM database but also simultaneously minimize the impacts of the de-identification on analysis.

## Discussion

### Principal Findings

The OMOP-CDM has already implemented a high level of anonymization through the ETL process. However, it is possible to re-identify an individual. Therefore, when analyzing the CDM database in a cloud-computing environment or other public spaces, additional personal information de-identification is required.

The results of applying the proposed de-identification strategy along with the k-anonymity, l-diversity, and t-closeness privacy models to each table, particularly to the “source value” fields of the OMOP-CDM database, indicate that the strongest anonymity could be achieved with the t-closeness model. Moreover, the l-diversity and t-closeness models have stronger anonymization criteria than k-anonymity; these models increase the size of the groups using the same identifier and quasi-identifier values to achieve their criteria. However, while the k-anonymity model alone effectively prevents re-identification in the CDM to some extent, the l-diversity and t-closeness models perform better in terms of protecting personal information.

Finally, considering the other databases, the size of the CDM database increases as the operating period increases. Therefore, de-identification of personal information should be periodically evaluated. Moreover, it is desirable to explore an appropriate privacy model and optimal conditions that suit the model.

### Conclusions

Although the OMOP-CDM has no identifier nor foreign key that can be linked to the institutional database during the ETL process, a risk of personal information exposure remains because it preserves some source values. Therefore, we proposed a de-identification strategy that establishes transformation rules (see Table 1) for privacy models such as k-anonymity, l-diversity, and t-closeness, for the OMOP-CDM schema; this strategy complies with the recommended security policy. For instance, if the “person_source_value” of the PERSON table is “1234567890,” it could be masked as “12345*****” after applying our de-identification strategy. This provides the flexibility to maintain the intentionally stored raw data. However, if the raw data exhibit a significantly low level of de-identification, it would be reasonable to not use the fields or tables. As a result of the evaluation of our de-identification strategy applied to the CDM database, it is possible to identify practical considerations for appropriate de-identification actions for each field. Thus, this research is a first step toward the development of safer and more appropriate de-identification policies for the OMOP-CDM schema and is expected to lay the foundation for further acceleration of CDM research.

## Abbreviations

DEA: Drug Enforcement Administration
ETL: extract-transform-load
HIPAA: Health Information Portability and Accountability Act
NLP: natural language processing
NPI: National Provider Identifier
OHDSI: Observational Health Data Sciences and Informatics
OMOP-CDM: Observational Medical Outcomes Partnership Common Data Model
